# Exploring Infant Fall Events Using Online Parenting Discussion Forums: Infodemiology Study

**DOI:** 10.2196/34413

**Published:** 2022-05-10

**Authors:** Nipuna Cooray, Si Louise Sun, Susan Adams, Lisa Keay, Natasha Nassar, Julie Brown

**Affiliations:** 1 The George Institute for Global Health Faculty of Medicine and Health UNSW Sydney Newtown Australia; 2 School of Women’s and Children’s Health UNSW Sydney Sydney Australia; 3 Department of Paediatric Surgery Sydney Children's Hospital Randwick Australia; 4 School of Optometry and Vision Science Faculty of Medicine and Health UNSW Sydney Sydney Australia; 5 Children’s Hospital at Westmead Clinical School Faculty of Medicine and Health University of Sydney Sydney Australia

**Keywords:** falls, child injury, online discussion forums

## Abstract

**Background:**

Falls represent the most common mechanism of injury requiring hospitalization among children under 12 months, and they commonly result in traumatic brain injury. Epidemiological studies exploring infant falls demonstrate the experienced burden, but they lack contextual information vital to the development of preventive interventions.

**Objective:**

The objective of this study was to examine contextual information for falls involving children under 12 months, using online parenting discussion forums.

**Methods:**

Online parenting forums provide an unobtrusive rich data source for collecting detailed information about fall events. Relevant discussions related to fall incidents were identified and downloaded using site-specific Google Search queries and a programming script. A qualitative descriptive approach was used to analyze the incidents and categorize contextual information into “precursor events” and “influencing factors” for infant falls.

**Results:**

We identified 461 infant fall incidents. Common fall mechanisms included falls from furniture, falls when being carried or supported by someone, falls from baby products, and falls on the same level. Across the spectrum of fall mechanisms, common precursor events were infant rolling off, infant being alone on furniture, product misuse, caretaker falling asleep while holding the infant, and caretaker tripping/slipping while carrying the infant. Common influencing factors were infant’s rapid motor development, lapses in caretaker attention, and trip hazards.

**Conclusions:**

The findings define targets for interventions to prevent infant falls and suggest that the most viable intervention approach may be to target parental behavior change. Online forums can provide rich information critical for preventive interventions aimed at changing behavior.

## Introduction

Injury is a widespread and longstanding public health problem [1]. Globally, injury is a leading cause of child death and hospitalization [2]. In Australia, children aged ≤1 year have the highest death rates due to injury among all children and have an injury hospitalization rate of 799/100,000 [3]. Falls are the most common injury mechanism in this age group, accounting for almost 50% of all injury hospitalizations [3]. The head is the most commonly injured body region [4], and head injury often leads to traumatic brain injury [5]. Similar incidences and injury patterns occur in North America and Europe [6-8]. Traumatic brain injury in early childhood is associated with negative behavioral and cognitive outcomes [9]. While a number of interventions are effective for minimizing fall risk in older children [10,11], there is a paucity of evidence on effective countermeasures for falls in children aged ≤1 year.

Epidemiologic studies examining infant falls usually rely on administrative data or medical records [4,12,13]. These provide details on burden and demographic risk factors, but generally have limited or incomplete contextual information. This is a barrier for effective intervention development [14].

The best source of detailed contextual information about infant falls is from someone who witnessed the fall. However, one-on-one discussions and large sample sizes can be time and resource intensive. In other areas, the internet and social media have been successfully used to collect data from people participating in online forum discussions [15]. These also provide naturalistic data, as discussions occur without researcher involvement [16].

We aimed to use online parenting discussion forums to unobtrusively and cost-effectively access contextual information about infant falls in order to identify specific modifiable factors to prevent infant falls.

## Methods

### Study Design

This was an infodemiology study [17,18] using online forum data following a qualitative descriptive approach [19], with the objective of providing comprehensive summaries of infant fall events [20]. The data source was social media forums within an online parenting website. This website was established in 1999, and is owned and operated by a large Australian media company. The website provides parenting information in the form of media articles and forums across a broad range of noninjury/prevention-related child care topics. After obtaining required approvals, site-specific Google Search queries were chosen to identify URLs potentially containing discussions related to infant falls. These were “baby fall,” “baby falling,” “baby fell,” “baby dropped,” and “baby hurt.” This approach allowed us to search all forums on this website without placing too much burden on the website. A researcher manually screened the resulting URL list and compiled a list of possible URLs containing infant fall discussions. The discussions from the selected list of web pages were downloaded using a program script written in Python, and any potentially identifiable data were removed. The search was completed on June 22, 2019, and included discussion threads ranging from November 22, 2003, to June 05, 2016.

Deidentified data were coded using QSR Nvivo12 software to identify relevant incidents. Relevant incidents were those relating to falls or near falls involving children aged ≤1 year, with age identified from words in the post (post specifically mentioned age as ≤1 year, post was from a forum specific to children aged 0-6 months or 6-12 months, fall incident was mentioned in response to other incidents describing infant falls where age 0-12 months was mentioned, or post had the words “newborn” or “tiny baby”).

### Ethics Approval

The study obtained approval from the website owner and ethics approval from the Human Research Ethics Committee (HC180295).

### Analysis

Fall mechanisms were categorized, described, and mapped to ICD-10-AM (International Statistical Classification of Diseases and Related Health Problems, Tenth Revision, Australian Modification) codes (Table 1). Two researchers (NC and SLS) independently coded the data using fall mechanism categories (Table 1), and any differences were discussed until agreement was reached.

Coding of contextual information followed an inductive open-coding approach. Emerging codes were then classified as “precursor events” or “influencing factors.”

In recognition that fall circumstances are often multilayered, we separated likely causative factors leading to the fall into “precursor events” and other “influencing factors.” A “precursor event” was defined as the event/state immediately before the fall according to the literal meaning in the discussion. An “influencing factor” was defined as a factor that impacted the “precursor event” and therefore the occurrence of the fall.

One researcher (NC) compiled a list of factors categorized as a “precursor event” or “influencing factor,” and a second researcher (SLS) independently recoded the data using this list, adding new factors as necessary. The two researchers (NC and SLS) compared analyses, and differences were discussed until agreement was reached. To address potential coder biases and preconceptions, coding for each fall mechanism by each researcher was undertaken separately, and consensus was reached before moving onto coding for the next mechanism.

NC (a PhD student) and SLS (an undergraduate medical student) were supervised by senior co-authors experienced in injury and qualitative research methods.

**Table 1:** Fall mechanism categories derived from ICD-10-AM (International Statistical Classification of Diseases and Related Health Problems, Tenth Revision, Australian Modification) codes.

Fall mechanism category and detailed fall mechanism			ICD-10-AM^a^ codes		ICD-10-AM code description
**Fall from household furniture**					
	Fall from bed	W06		Fall from bed	
	Fall from chair/couch/sofa	W07		Fall from chair	
	Fall from changing table	W08		Fall from other furniture	
	Fall from table	W08		Fall from other furniture	
**Fall from baby products**					
	Fall from baby capsules	W08		Fall from other furniture	
	Fall from bassinet/cot	W08		Fall from other furniture	
	Fall from bouncer	W08		Fall from other furniture	
	Fall from child car restraints	W08		Fall from other furniture	
	Fall from high chair/baby chair	W08		Fall from other furniture	
	Fall from portable baby bed	W08		Fall from other furniture	
	Fall from pram/stroller	W08		Fall from other furniture	
**Fall while being carried or supported by someone**					
	Fall when carried or supported by mother	W04		Fall while being carried or supported by other persons	
	Fall when carried or supported by an unidentifiable parent	W04		Fall while being carried or supported by other persons	
	Fall when carried or supported by an adult caretaker (other than parents)	W04		Fall while being carried or supported by other persons	
	Fall when carried or supported by an older child	W04		Fall while being carried or supported by other persons	
**Fall on the same level**					
	Fall while infant standing	W01		Fall on the same level from slipping, tripping, and stumbling	
	Fall while infant sitting	W18		Other fall on the same level	
	Fall while infant crawling	W18		Other fall on the same level	
	Other fall on the same level due to collision	W03		Other fall on the same level due to collision with or pushing by another person	
**Fall related to stairs**					
	Fall on and from stairs and steps	W10		Fall on and from stairs and steps	
**Fall between levels**					
	Fall from, out of, or through building or structure	W13		Fall from, out of, or through building or structure	
	Fall from a cliff	W15		Fall from a cliff	
	Other fall from one level to another	W17		Other fall from one level to another	
**Other fall mechanisms**					
	Fall from mats or playmats	W08		Fall from other furniture	
	Fall involving play equipment	W09		Fall involving playground equipment	
	Fall from shopping cart	W08		Fall from other furniture	

^a^ICD-10-AM: International Statistical Classification of Diseases and Related Health Problems, Tenth Revision, Australian Modification.

## Results

### Overview

**Figure 1:**
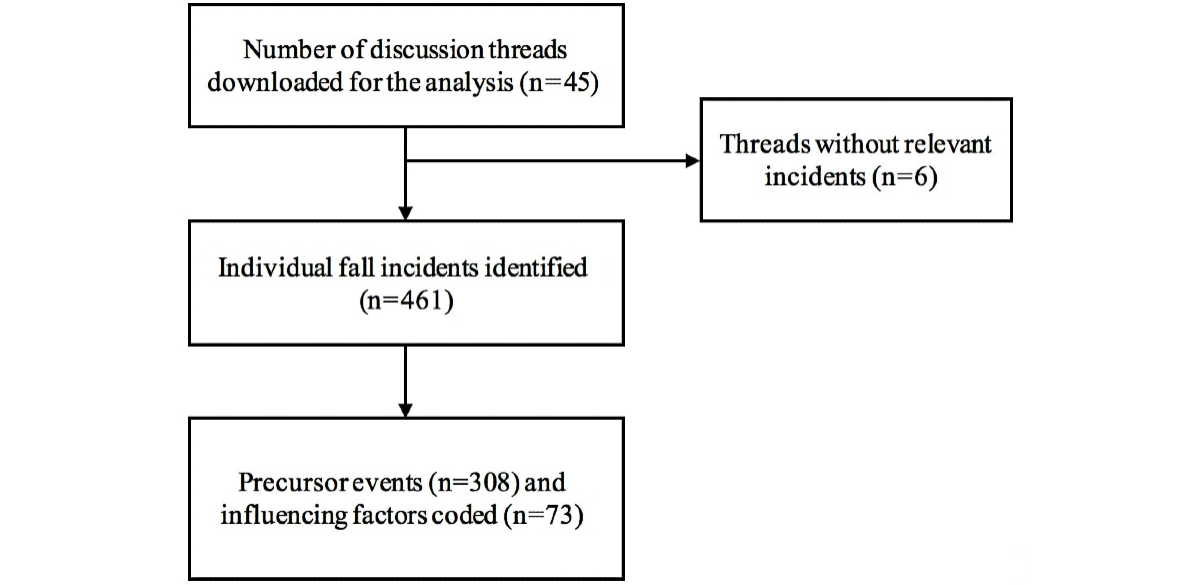
Outcome of the data capturing process.

Figure 1 summarizes the data capture process. Overall, 461 infant fall incidents were identified. The most common fall mechanisms were a fall from household furniture (270/461, 58.6%), followed by falls when being carried or supported by someone (92/461, 20.0%) and falls from baby products (55/461, 11.9%). Other mechanisms were a fall on the same level (28/461, 6.1%), fall on/from stairs (6/461, 1.3%), falls from playmats (4/461, 0.9%), falls from playground equipment (3/461, 0.7%), and falls from shopping carts (3/461, 0.7%).

### Falls From Household Furniture

Detailed mechanisms for these falls included falls from beds (146/270, 54.1%), falls from changing tables (64/270, 23.7%), falls from chairs/couches/sofas (53/270, 19.6%), and falls from tables (6/270, 2.2%).

The most commonly mentioned precursor event for falls from household furniture was the infant rolling off the furniture. This was mentioned 71 times within the 270 (26.3%) incidents related to furniture falls.

Yesterday, my little girl (6 months) rolled off the bed. She hit her head … and screamed…

The next most common precursor event involved the infant being left alone on furniture. This was mentioned in 36 of the 270 (13.3%) incidents.

I left her in the middle of my queen bed while I did some vacuuming. As I got closer to my bedroom … I could hear her screaming like she had never screamed before. I ran into the room and she was on the floor!...

The caretaker falling asleep with the baby was another common precursor event mentioned in 18 of the 270 (6.7%) incidents.

… I was breastfeeding him in bed and fell asleep with him on the outside. I woke up when I heard a thud and DS[Darling Son] cry.

It was clear from some discussions that the precursor event of falling asleep was often unintentional (10/18, 55.6%), while in others (6/18, 33.3%), it was intentional or the intention was unclear (2/18, 11.1%).

For falls from changing tables, a common precursor event was the caretaker reaching for something while nappy changing, which was identified 11 times within the 64 (17.2%) incidents related to changing tables.

I was changing him on the change table and all I did was slip one hand down to put the dirty nappy in the nappy bag and ds launched himself off the table and landed on the floor…

Unexpected or rapid changes in motor development were the most common influencing factor for furniture falls. This was identified in 29 of the 270 (10.7%) incidents.

…when he had started to move - I underestimated how quick he was. I used to put him on our bed every morning while I got dressed. One day I turned my back for a second and in that time he pulled himself to the edge then did a somersault off the bed!

Lapse in caretaker attention was the next most common influencing factor for this fall mechanism. This was identified in 20 of the 270 (7.4%) incidents.

… honestly it can happen in the blink of an eye. Similar to your DH[Darling Husband] I looked away from the table, and over he went. It was so quick.

### Falls When Carried or Supported by Someone

This was the second most common fall mechanism in the discussions (n=92). It commonly occurred when the child was carried or supported by the mother (39/92, 42.4%) or an unidentified parent (28/92, 30.4%), and when the child was carried or supported by an adult caretaker other than a parent (19/92, 20.7%), and less commonly occurred when the child was carried or supported by an older child (6/92, 6.5%).

The most common precursor for these falls was the caretaker tripping/slipping (29/92, 31.5%), and this often occurred on steps or stairs (18/29, 62.1%). Other environmental hazards within the home included slippery floors (2/29, 6.9%) and tripping hazards on the floor (2/29, 6.9%).

I dropped my ds[Darling Son] he was about 10 months tripped up the back step he screamed has a giant bump…

Another common precursor event for these falls was the person falling asleep while holding the infant (15/92, 16.3%), and it often involved the child’s mother falling asleep while feeding (12/15, 80.0%).

…I was totally sleep deprived. Sat down on the couch to nurse her, dozed off with her snuggled low in my arms (basically in my lap) our dog barked and I startled awake – DD[Darling Daughter] rolled down my legs and into the coffee table.

A tired caretaker is also a likely influencing factor; however, this was only overtly discussed a few times (2/15, 13.3%). Other commonly discussed influencing factors were inadequate holding of the child (13/92, 14.1%) and sudden unexpected movements of the child (9/92, 9.8%).

A friend was holding my 6 month old he had his arm tucked behind his legs holding him up right and wasn't supporting his back when my LO flung back… (“LO” is assumed to mean “little one”)

### Falls From Baby Products

The most common products involved in falls were strollers/prams (21/55, 38.2%), bouncers (10/55, 18.2%), high chairs/baby chairs (9/55, 16.4%), and bassinets/cots (9/55, 16.4%). Less commonly involved were baby carriers/capsules (3/55, 5.5%), child car restraints (2/55, 3.6%), and portable baby beds (1/55, 1.8%).

Improper use was the most common precursor event for these falls. Nonuse or misuse of safety straps was common for many baby products (particularly for strollers/prams, baby bouncers, high chairs/baby chairs, child car restraints, and baby capsules/carriers). This was identified 30 times (55%, 15 cases of not using safety straps and 15 cases of apparent improper use of straps).

Mother of the Year here took a few months to really internalise the 'strap them in' message and DD[Darling Daughter]1 bounced herself face first out of the bouncer at about three months old

Some other critical misuses identified were placing the cot base in a high position (5/55, 9.1%), unbalancing the stroller (3/55, 5.5%), not using strollers’ brakes (2/55, 3.6%), and carrying the infant while in the bouncer/portable baby bed (2/55, 3.6%). Falls from cots were influenced by rapid motor development.

…like he was balancing on the cot railing with his feet off the mattress suspended in mid air by piece of wood…

### Falls on the Same Level

Four different types of falls on the same level were mentioned. Most common was a fall while the infant was standing (20/28, 71.4%). Falls while the infant was sitting, falls while the infant was crawling, and other falls due to being pushed by another person had less than five identified incidents each.

The common influencing factor for this fall mechanism was the child’s underdeveloped motor skills (13/28, 46.4%).

…Now that both are easily pulling themselves up against furniture to stand, they are doing it every chance they get. The only problem is once they get up they don't know how to get down or lose concentration, let go and fall...a lot of the time hitting their heads on the tiles.

### Falls on or From Stairs

Falls on or from stairs were relatively uncommon (5 incidents). Two influencing factors for these were lapses in caretaker attention (3/5, 60%) and unexpected/rapid infant motor development (2/5, 40%).

DS[Darling Son]1 fell down the stairs - all 8 of them - when he was 4 months. He was lying at one end of the room, well away from the stairs. I put a book on the shelf and when I turned back he'd rolled across the room and I was just in time to see him disappear, screaming, down the stair well.

### Other Fall Mechanisms

Other mechanisms identified from the discussions included falls from playground equipment and falls from shopping carts (10 incidents). The precursor event related to falls from shopping carts was the nonuse of straps (3/10, 30%).

…didn't bother to buckle him in. I was squatting down looking at something when I heard a horrible splat sound, he had fallen face first onto the cement floor…

There were no detailed discussions to identify causal factors for falls from playground equipment. Moreover, there were no discussions of falls between levels (eg, from windows).

Table 2 summarizes the precursor events and influencing factors for different fall mechanisms.

**Table 2:** Precursor events and influencing factors for fall mechanisms.

Fall mechanism	Precursor events	Influencing factors
Fall from furniture	Infant rolling off Infant being left alone on furniture Caretaker falling asleep with the infant Reaching for something while nappy changing	Unexpected or rapid changes in infant motor development Lapse in caretaker attention
Fall when carried or supported by someone	Caretaker tripping or slipping Caretaker falling asleep while holding the infant	Inadequate holding of the child Sudden unexpected movement of the infant
Fall from baby products	Nonuse or misuse of safety straps Other product misuses	N/A^a^
Fall on the same level	N/A	Infant’s underdeveloped motor skills
Fall on or from stairs	N/A	Lapse in caretaker attention Unexpected or rapid changes in infant motor development

^a^N/A: not applicable.

## Discussion

### Principal Findings

Using a novel qualitative infodemiological approach, we identified contexts requiring intervention to prevent the majority of falls in children aged ≤1 year. These are leaving children alone on furniture; misuse of changing tables and baby products, such as strollers, baby carriers, and baby chairs; slips and trips; and falling asleep while holding an infant. Furthermore, the richness of our data set allowed us to link specific influencing factors to specific precursor events for these fall types to identify modifiable factors to prevent falls. These include awareness of unexpected or rapid changes in infant motor development, lapses in caretaker attention, importance of adequately holding the infant, and reducing hazards in the home environment.

Our findings align well with previously reported studies using administrative data sets and medical record reviews [4,7,21-24]. While some identified factors have been noted previously [8,25-27], this is the first study to provide this level of detail and identify targets for intervention across the spectrum of fall mechanisms among infants of this age.

Contextual information like that identified in this work also provides evidence and adds to studies that have previously suggested using age appropriate injury prevention education for caregivers and home safety assessment programs [7,25]. However, currently, evidence on any effective interventions specifically targeting falls in this age group is rare [28]. Given the magnitude and potential impact of this problem [5,8], there is an urgent need to fill this gap and identify effective targeted interventions. The outcomes of this work identify modifiable factors to be targeted in these interventions.

While our findings demonstrate that no single intervention would prevent all falls, there is a common need for parent/caretaker behavioral change across many of the fall mechanisms. It therefore appears that a behavior change or an active approach, rather than a purely structural change (passive approach) [29], may be effective to prevent infant falls. For example, having a safety harness in a changing table is structural, whereas the parent using it appropriately is behavioral. However, behavior change is complex, and educational interventions alone usually do not enact behaviors [30]. Behavior change interventions are more likely to be successful when based on behavior theory [31]. One challenge to developing effective behavior change interventions is that they require detailed understanding of the problem and target behaviors [32]. This study fills some of these gaps by identifying behaviors that need to change, and the circumstances where these behaviors occur.

In this study, we did not attempt to examine data by infant age, but it is clear from our earlier work [4] that risks of falls by different mechanisms change as children move through development stages in the first year of life. Different behavior change interventions are likely needed at different times through this year, and this needs to align with the infant’s developmental stage [6]. For example it is possible that the risk of a mother falling asleep while feeding is higher in early infancy and the risk of rolling off the bed is higher when the infant is gaining motor skills. Intervening at a single time point may also not be as effective as a targeted strategy to deliver behavior change interventions at different time points over time.

Falls among infants on the same level were discussed relatively less commonly by parents in the forums than other falls, yet it is likely that these occur very commonly. As noted by Adolph and Berger [33], falling is a common by-product of children learning to walk, with children at this stage of development falling within the vicinity of 17 times an hour and 100 times a day. As we previously observed [4], these types of falls very rarely occur among infants hospitalized from a fall (<2% of all patients), and therefore, this lack of severity might underpin the lack of discussion in the forums. As these falls occur while children are developing an important motor skill, it would not be appropriate to try to prevent the activities leading to these. Instead, injury risk might best be reduced by paying attention to the environment in which children are placed during this stage of development.

Another aspect warranting further environmental examination is the adequacy of both the design and instructions of common baby products used by parents of infants. Previous work identifying the influence of design defects in products, such as prams/strollers [34,35] and high chairs [34,36], has led to stringent safety standards. However, these types of design standards do not address how the products are ultimately used. Improper use was the most common precursor event for falls involving baby products. This aligns with findings from previous studies reporting the high frequency of nonuse or incorrect use of safety straps in products, such as prams/strollers and high chairs [34,37]. In other areas (eg, child car seats), it is becoming increasingly clear that correct use requires attention to how usage information is communicated and the interaction between the user and the inherent design of the product, in addition to the general behavior of the user [38]. Extension of this approach to all baby products may be useful.

### Limitations

As this is a qualitative study based on ad hoc reporting of fall types, the frequencies of different fall types reported might not reflect true frequencies. Frequencies are reported to give readers some idea of the commonality, mechanisms, influencing factors, and precursor events. While care was taken when extracting data to exclude conversations in separate threads related to the same fall incident, this could not be guaranteed. Therefore, this might also impact the accuracy of specific fall mechanisms reported. However, common fall mechanisms aligned with other epidemiological and medical record reviews [4,7,22]. Data used were from a convenience sample of online forum discussion participants, and the sample characteristics are unknown. The characteristics of parents who use social media are not well understood in terms of how well they represent the full population of parents or their behavior in discussing injury events of different severities online. Therefore, this may introduce some unknown bias, and the findings may not be generalizable to the whole population or the full spectrum of injury severity. Data were also collected across a broad time period of 13 years, and there is no way to know the specific geographical locations of those contributing to the forum from which the data were collected. While to our knowledge, there were no significant changes in health promotion/injury prevention programs across this time period, it is possible that contributors were exposed to different types of health promotion activities depending on location. This may have also introduced some unmeasured bias in the data. Another limitation was the use of a single search engine. Different search engines may provide different result sets. Moreover, this kind of study collects data from a static point in time, which precludes active engagement with caregivers and the ability to clarify or obtain additional details from parents compared with other qualitative approaches. However, the unobtrusive nature of this data collection method may be a strength, as it provides data extracted from naturalistic parental discussions.

### Conclusion

This study used infant fall incidents from online parenting forums to identify precursor events and influencing factors leading to different fall types among infants aged ≤1 year. This information is paramount to the development of preventive interventions, particularly given that the findings suggest targeting parental behavior.
